# Efficacy and safety of intrapleural perfusion with hyperthermic chemotherapy for malignant pleural effusion: a meta-analysis

**DOI:** 10.1186/s13019-024-02751-6

**Published:** 2024-05-06

**Authors:** Xue Pan, Zhichao Hou, Tangjuan Zhang, Zheng Ding, Fei Ye, Zhulin Wang, Chunyao Huang, Peng Wang, Xiangnan Li

**Affiliations:** 1 School of Nursing and Health, Zhengzhou University, Zhengzhou, 450001 China; 2 Department of Thoracic Surgery, The First Affiliated Hospital of Zhengzhou University, Zhengzhou, 450052 China; 3 Department of Emergency, The First Affiliated Hospital of Zhengzhou University, Zhengzhou, 450052 China

**Keywords:** Systematic review, Efficacy, Safety, Malignant pleural effusion, Hyperthermic perfusion chemotherapy

## Abstract

**Objective:**

To evaluate the efficacy and safety of intrapleural perfusion with hyperthermic chemotherapy (IPHC) in treating malignant pleural effusion (MPE).

**Methods:**

PubMed, Embase, Cochrane Library, Chinese National Knowledge Infrastructure (CNKI), Chinese Biomedical Literature Database (CBM), VIP Chinese Science and Technology Journal Full-text Database (VP-CSJFD), and Wanfang database were searched by computer from database establishment to January 17, 2024. Relevant randomized controlled articles with IPHC as the observational group and intrapleural perfusion chemotherapy (IPC) as the control group for MPE were included. Then, the methodological quality of the included articles was evaluated and statistically analyzed using Stata 16.0.

**Results:**

Sixteen trials with 647 patients receiving IPHC and 661 patients receiving IPC were included. The meta-analysis found that MPE patients in the IPHC group had a more significant objective response rate [RR = 1.31, 95%CI (1.23, 1.38), *P* < 0.05] and life quality improvement rate [RR = 2.88, 95%CI (1.95, 4.24), *P* < 0.05] than those in the IPC group. IPHC and IPC for MPE patients had similar incidence rates of asthenia, thrombocytopenia, hepatic impairment, and leukopenia.

**Conclusion:**

Compared with IPC, IPHC has a higher objective response rate without significantly increasing adverse reactions. Therefore, IPHC is effective and safe. However, this study is limited by the quality of the literature. Therefore, more high-quality, multi-center, large-sample, rigorously designed randomized controlled clinical studies are still needed for verification and evaluation.

## Introduction

Malignant pleural effusion (MPE) is one of the most common side effects of malignant tumors. Cancer cells may be found in the patient’s pleural effusion, which is mainly caused by the primary pleural malignant tumors or the metastasis of malignant tumors at other sites to the pleura. Dry cough, chest pain, progressively worsening shortness of breath, and dyspnea are the main manifestations of MPE. According to the research conducted in the United States, there are over 150,000 new cases of MPE per year [1]. Patients with advanced lung cancer are often accompanied by MPE, which is classified as M1a in the TNM classification of lung cancer (eighth version) and indicates a worse prognosis [2]. The survival of patients with MPE ranges from 3 to 12 months, and the 30-day mortality rate is 29–50% [3,4]. The therapeutic effect and prognosis of cancer patients are affected by MPE. Currently, pleurodesis using minocycline, OK-432, or talc for treating MPE was reported in the literature, and the success rate of pleurodesis was about 64% [5]. The result of that treatment needs to be more satisfactory. Besides, MPE is treated with systemic chemotherapy, intrathoracic chemotherapy, and drainage of pleural effusions, and they often function to relieve symptoms, ease pain, or improve patients’ quality of life [6]. Most malignancies with MPE respond poorly to systemic chemotherapy [7]. Draining pleural effusions is the most widely used treatment for MPE. However, the effusions still recur rapidly even when sclerosing agents or anticancer drugs are injected into the thoracic cavity [8–10]. Intrapleural perfusion chemotherapy (IPC) kills tumor cells at the pleural site by injecting chemotherapeutic medicines into the thoracic space. Wallner et al. [11] and Hettinga JV et al. [12] reported that heating cisplatin perfused into the thoracic cavity to 43℃ effectively killed tumor cells sensitive to cisplatin, suggesting that a certain degree of heating could improve the cytotoxic response of cisplatin. Intrapleural perfusion with hyperthermic chemotherapy (IPHC), a type of local thoracic chemotherapy, kills tumor cells by combining hyperthermia and regional chemotherapy. The closed circulation system built into the extracorporeal circulation system circulates medications in the pleural cavity at the right temperature (43–45 °C) during the local thoracic chemotherapy [6,13].

IPC therapy has the advantages of simple operation and patient tolerance, and thus, it is widely used in clinical practice. The IPHC therapy requires thermal action to participate in this treatment, which is inconvenient, and in addition, it is essentially hyperthermia so patients must tolerate it. Although the IPHC improves the efficacy of chemotherapy drugs through thermal action, it is still unknown whether it enhances their toxic side effects. There still needs to be high-level evidence-based medical evidence to demonstrate the effectiveness and safety of IPHC. Therefore, this paper mainly evaluated the two therapeutic methods of IPC and IPHC through meta-analysis, providing some reference value for MPE treatment in clinical practice.

## Materials and methods

### Literature search

To gather the literature on IPHC for treating MPE, two researchers independently searched the Chinese National Knowledge Infrastructure (CKNI), VIP Chinese Science and Technology Journal Full-text Database (VP-CSJFD), Chinese Biomedical Literature (CBM), Wanfang Data Journal Article Resource (WangFang), PubMed, The Cochrane Library, and Embase from database establishment to January 17, 2024, based on the search criteria of each database. The retrieved languages were limited to Chinese and English. Search terms were malignant pleural effusion, carcinomatous pleural effusion, pleural thermal perfusion, intrapleural chemotherapy, intrapleural perfusion, and hyperthermic chemotherapy. The search method was a combination of subject terms and free words, and the search formula was: ((Malignant pleural effusion) OR (Carcinomatous pleural effusion)) AND ((((Pleural thermal perfusion) OR (Intrapleural chemotherapy)) OR (intrapleural perfusion)) OR (hyperthermic chemotherapy)).

### Inclusion and exclusion criteria

Inclusion criteria: (1) Patients having moderate to a large amount of pleural effusion where cancer cells were found, which were confirmed by pathological examination and CT or color doppler ultrasound; (2) The control group was given IPC, and the observation group was given IPHC; (3) Outcome indicators included objective response rate, improvement of quality of life, adverse reaction (Asthenia, Thrombocytopenia, Hepatic, Chest pain, Leukopenia, Gastrointestinal Reactions), (4) The study type of the literature was a randomized controlled trial (RCT).

Exclusion criteria: (1) There is no controlled trial; (2) There are insufficient or complex data in the literature; (3) literature review, case report, and meta-analysis; (4) Repeated published literature; (5) The observation group using reperfusion after heating in addition to chemotherapeutic drugs or circulatory perfusion using a heating device.

### Literature screening and data extraction

(1) The obtained articles were imported into the EndnoteX9 program. Two researchers excluded the unqualified articles by initially reviewing the titles and abstracts according to the inclusion and exclusion criteria after deleting the redundant literature. Subsequently, two researchers evaluated the remaining literature thoroughly and comprehensively and removed articles with incomplete outcome measurements, insufficient data, or duplicate data findings. (2) Two researchers collected the pertinent data, including the first author, publication year, publishing nation, sample size, age, gender, objective response rate, quality of life, and improvement (Asthenia, Thrombocytopenia, Hepatic, Chest pain, Leukopenia, Gastrointestinal Reactions). Among them, quality of life improvement is an increase of 10 points or more. After extracting and enhancing the data, two researchers integrated and checked the data, respectively. If there is a dispute, external experts with extensive knowledge of evidence-based medicine will be consulted to reach a decision together. The professional opinion of the third party will determine the outcome.

### Literature quality evaluation

The quality of the included randomized controlled trials was evaluated using the Jadad scale. The following assessment criteria were used: random sequence creation, randomization concealment, blind technique, withdrawal, and loss of follow-up. On this scale, a score of (1–3) denotes poor literature, whereas a score of (4–7) denotes excellent literature.

The Newcastle-Ottawa Scale (NOS) is used to assess the quality of non-randomized studies, particularly cohort and case-control studies. A score of 5 or above indicates high quality in the literature.

### Statistical methods

Stata 16.0 was used to analyze the data and create forest and funnel plots. The relative risk (RR) and its 95% confidence interval (CI) were used to represent the effect magnitude of the enumeration data. In statistics, *P* < 0.05 indicates a significant difference. Heterogeneity test criteria: When I^2^ < 50% and *P* > 0.10 showed less heterogeneity, the fixed-effect model was used; when I^2^ ≥ 50% and *P* < 0.10 indicated more heterogeneity, the random effect model was used. Publication bias is one of the most common system errors in Meta-analysis. When the number of included studies was more than or equal to 10, the symmetry of the funnel plot could be visually inspected for the publication bias test. When the number of included literature for each outcome measure was within the range of 2–10, the publication bias of outcome measures could not be accurately assessed through the funnel. Stata16.0 software was employed to evaluate the publication bias among the included studies by the Egger test, and *P* > 0.05 indicated no publication bias.

## Results

### The flow chart of literature retrieval and results

**Fig. 1:**
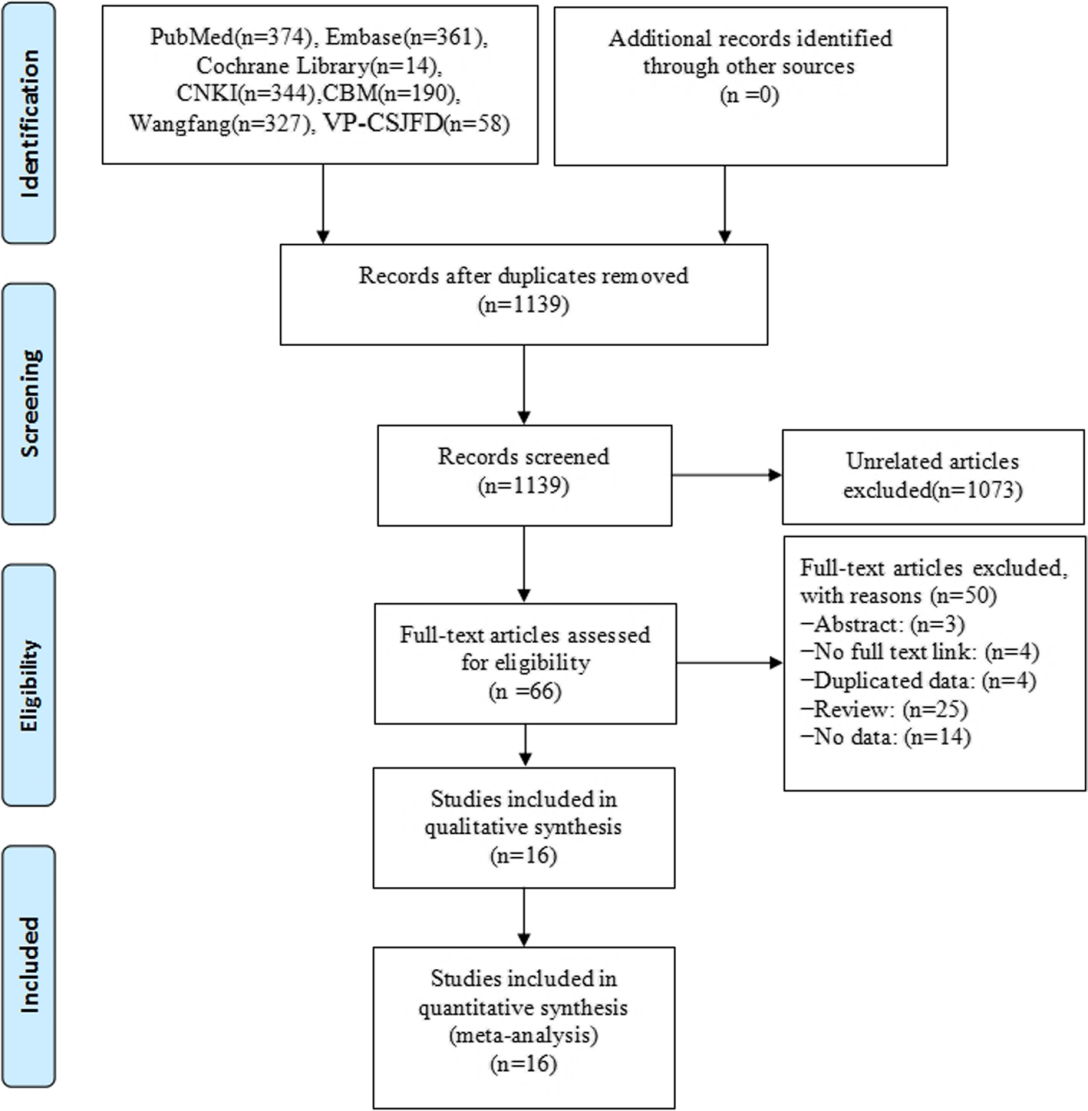
Literature screening flow chart

In this meta-analysis, 1668 relevant initial publications were discovered, consisting of 749 English papers and 919 Chinese ones. 16 reports were included after reading their titles and abstracts and evaluating them for inclusion and exclusion. 1308 patients from 16 articles [14–29] were evaluated (Fig. 1).

### Basic characteristics and quality evaluation of the included literature

This meta-analysis included 14 randomized controlled trials and 2 cohort studies. All from Chinese scholars. Table 1 displays the comprehensive basic characteristics of the included literature. The randomized control concept was used in all of the included RCTs. Only three RCTs provided a more in-depth description of the particular randomization procedure. The blind method was not fully described in any of the included RCTs. Still, all the included RCTs had complete data without loss of follow-up or withdrawal. The Jadad score was only 3 [18,21,24] in the 3 included RCTs and ≥ 4 in the other RCTs. The NOS scores of the two cohort studies were both 5. Therefore, the overall quality of the articles was fair (Table 1).

**Table 1:** Basic characteristics and quality evaluation results of included literature

Author	Year	Country	Study type	Intervention		Sample size		Age(s)		Sex(M/F)		Disease	Heat-perfusion chemotherapy regimens	Objective Response Rate determination criteria	Combination therapy	Karnofsky Performance Status (KPS) scoring criteria	Courses of treatments	Origins of pleural effusion	Follow-up	Outcomes	Jadad/NOS scores
				OG	CG	OG	CG	OG	CG	OG	CG										
Yangfan Liu [14]	2016	China	RCT	THPC	IPC	42	42	65.38 ± 7.28	63.98 ± 6.18	29/13	26/16	MPE	Cisplatin, 50 mg / m^2^,43℃, 3 times per week	ORR = (CR + PR) / Total number of cases × 100%	NA	NA	1week	Non-small cell carcinoma	At least 4 weeks	ORR、Adverse reaction	4
Yangfan Liu^[[ [15]]^	2020	China	RCT	THPC	IPC	44	44	66.34 ± 4.26	66.31 ± 4.28	32/12	30/14	MPE	Cisplatin, 50 mg / m^2^,43℃, once / week	ORR = (CR + PR) / Total number of cases × 100%	NA	NA	4weeks	A variety of cancers	At least 4 weeks	ORR	5
Meiling Liang [16]	2016	China	RCT	THPC	IPC	28	28	NA	NA	NA	NA	MPE	Cisplatin, 60 mg / m^2^,43℃, once / week	ORR = (CR + PR) / Total number of cases × 100%	NA	An increase of > 10 points in Karnofsky score is considered improvement.	4weeks	Small cell lung cancer	At least 4 weeks	ORR、Adverse reaction、IQL	4
Chunlei Zhao [17]	2015	China	RCT	THPC	IPC	30	30	NA	NA	NA	NA	MPE	Conlyte heat perfusion, 42℃, 3 times per week	ORR = (CR + PR) / Total number of cases × 100%	NA	NA	2weeks	A variety of cancers	At least 4 weeks	ORR	4
Xuexin Xu [18]	2011	China	RCT	THPC	IPC	33	30	NA	NA	18/15	20/10	MPE	Conlyte heat perfusion, 43 ~ 45℃, once / week	ORR = (CR + PR) / Total number of cases × 100%	NA	An increase of > 10 points in Karnofsky score is considered improvement.	3weeks	A variety of cancers	At least 4 weeks	ORR、Adverse reaction、IQL	3
Chengyan Wu [19]	2017	China	RCT	THPC	IPC	31	31	60.4 ± 13.5	59.7 ± 12.8	24/7	23/8	MPE	Nedaplatin 40 mg / m^2^, 46℃, two times / week	ORR = (CR + PR) / Total number of cases × 100%	NA	NA	3weeks	Lung cancer	At least 4 weeks	ORR、Adverse reaction	4
Chaofeng Wang [20]	2020	China	RCT	THPC	IPC	28	28	NA	NA	14/14	15/13	MPE	Cisplatin 80 mg / m^2^,43℃, 3d / time	ORR = (CR + PR) / Total number of cases × 100%	NA	NA	2weeks	Non-small cell carcinoma	At least 4 weeks	ORR	4
Fengying Duan [21]	2003	China	RCT	THPC	IPC	12	14	55 ± 15	57 ± 13	8/4	9/5	MPE	Cisplatin from 60 to 80 mg / m^2^,45 to 47℃, 2 times / week	ORR = (CR + PR) / Total number of cases × 100%	NA	An increase of > 10 points in Karnofsky score is considered improvement.	3weeks	A variety of cancers	At least 4 weeks	ORR、Adverse reaction、IQL	3
Zhiwen Lu [22]	2013	China	RCT	THPC	IPC	19	19	52.3 ± 9.8		16/22		MPE	Cisplatin, 80 mg / m^2^,43 to 45℃, 2 times / week	ORR = (CR + PR) / Total number of cases × 100%	Rehydration、Hydration、Diuresis	An increase of > 10 points in Karnofsky score is considered improvement.	Unclear	A variety of cancers	At least 4 weeks	ORR、IQL	4
Zhibin Li [23]	2010	China	RCT	THPC	IPC	30	30	48	49	18/12	22/8	MPE	Cisplatin, 80 mg / m^2^,41 to 43℃, 2 times / week	ORR = (CR + PR) / Total number of cases × 100%	Rehydration、Hydration、Diuresis	An increase of > 10 points in Karnofsky score is considered improvement.	3weeks	A variety of cancers	At least 4 weeks	ORR、Adverse reaction、IQL	4
Guorong Yan [24]	2006	China	RCT	THPC	IPC	23	23	65	65.8	10/13	11/12	MPE	10 mg of mitomycin, 1000 mg of 5-fluorourea density, 40 mg of cisplatin, 42 to 43℃, 2 times per week	ORR = (CR + PR) / Total number of cases × 100%	NA	NA	3weeks	Unclear	At least 4 weeks	ORR	3
Lili Liu [25]	2019	China	RCT	THPC	IPC	44	44	63.41 ± 8.87	63.26 ± 9.15	23/2l	24/20	MPE	Cisplatin, 60–100 mg / m^2^,43℃, 2 times / week	ORR = (CR + PR) / Total number of cases × 100%	NA	NA	2weeks	Non-small cell carcinoma	At least 4 weeks	ORR、Adverse reaction	5
Yejun Cao [26]	2021	China	RCT	THPC	IPC	10	27	NA	NA	5/5	17/10	MPE	Platinum-based chemotherapy agents, 80 mg / m^2^,43℃	ORR = (CR + PR) / Total number of cases × 100%	Rehydration、Hydration、Diuresis	NA	Unclear	Unclear	At least 4 weeks	ORR、Adverse reaction	5
Wen-Jun Chen [27]	2012	China	RCT	THPC	IPC	165	163	56.7 ± 11.3	43.3 ± 10.41	91/74	88/75	MPE	Cisplatin, from 40 to 60 mg / m^2^	ORR = (CR + PR) / Total number of cases × 100%	Systemic chemotherapy	NA	8weeks	Small cell lung cancer	At least 4 weeks	ORR	4
Dong Cao [28]	2023	China	Cohort Study	THPC	IPC	48	48	56.44 ± 5.51	56.42 ± 5.52	30/18	29/19	MPE	Cisplatin 70 mg / m^2^,43℃, 3d / time	ORR = (CR + PR) / Total number of cases × 100%	Comprehensive intervention	NA	2weeks	Non-small cell carcinoma	At least 4 weeks	ORR、Adverse reaction	5
Yi Cao [29]	2022	China	Cohort Study	THPC	IPC	60	60	47 ~ 72	50 ~ 75	NA	NA	MPE	Cisplatin 200 mg / m^2^,43℃	ORR = (CR + PR) / Total number of cases × 100%	NA	NA	Unclear	A variety of cancers	At least 4 weeks	ORR、Adverse reaction	5

*OG* Observation group, *CG* Control group, *RCT* randomized controlled trial, *THPC* thoracic hyperthermic perfusion chemotherapy, *IPC* intrapleural chemotherapy, *ORR* Objective response rate, *ORR* (CR + PR) / Total number of cases × 100%, *NA* unavailable, *MPE* Malignant pleural effusion, *M* male, *F* female, *IQL* Improvement of quality of life. CR (Complete Response): Patient symptoms disappear, effusion completely resolves, and condition remains stable for over 4 weeks; PR (Partial Response): Patient symptoms improve, effusion reduces by ≥ 50%, and no increase for a continuous 4 weeks. KPS scoring criteria: Physical status assessment follows the Karnofsky Performance Scale. An increase of > 10 points in the Karnofsky score is considered an improvement, a change of < 10 points in the Karnofsky score is considered stable, and a decrease of ≥ 10 points in the Karnofsky score indicates a decline

### Meta-analysis results

#### Objective response rate

**Fig. 2:**
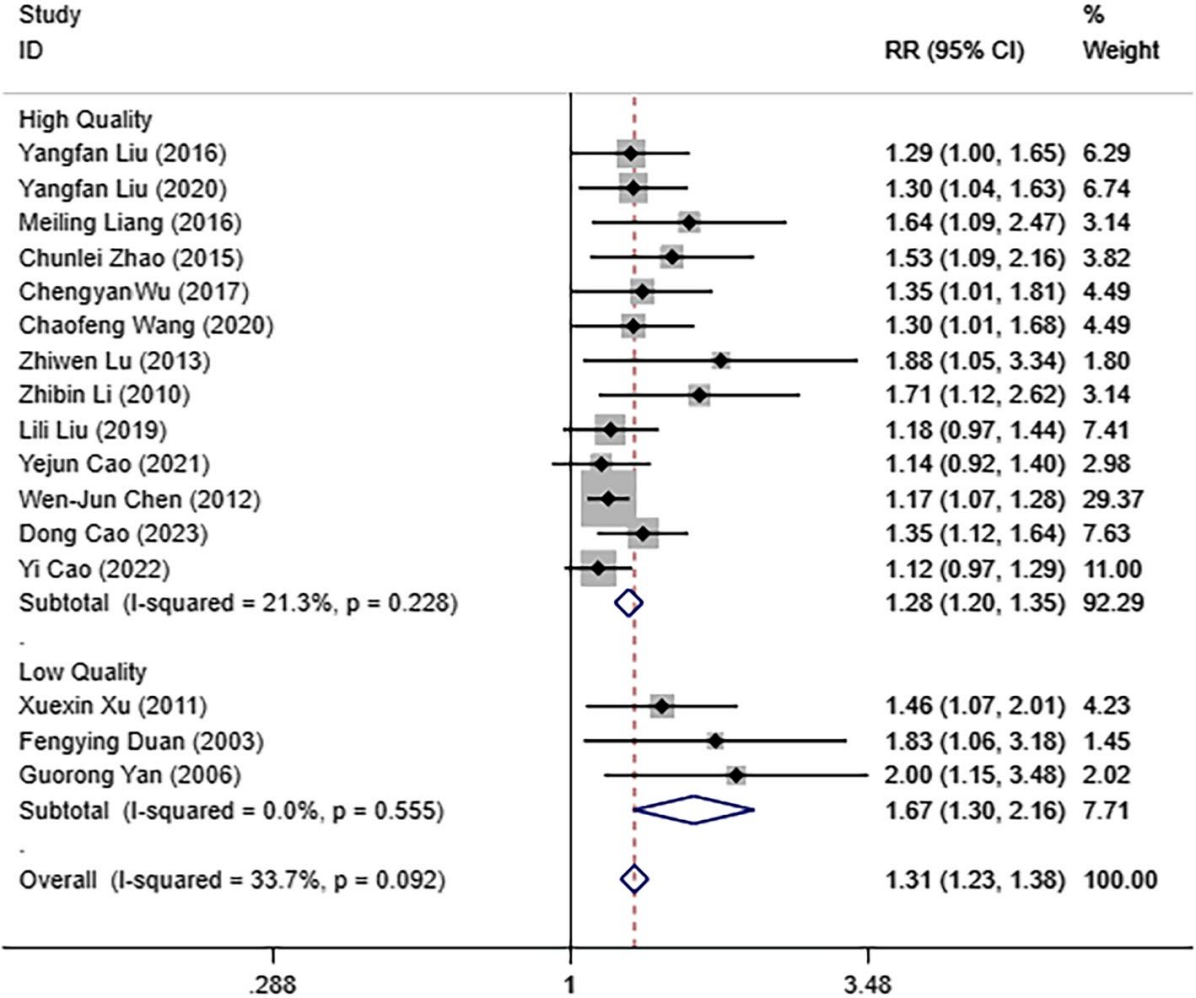
Forest plot for comparison of objective response rate for MPE patients in IPHC and IPC groups based on the literature quality subgroup analysis. Note: MPE, malignant pleural effusion; IPHC, intrapleural perfusion with hyperthermic chemotherapy; IPC, intrapleural perfusion chemotherapy

The objective response rate (ORR) after IPHC or IPC was obtained from 16 studies [14–29], where 641 MPE patients underwent IPHC and 661 patients with IPC. There is little heterogeneity among the analyzed studies, according to the results of the heterogeneity analysis for this literature (I^2^ = 33.7% and *P* = 0.092). The results were combined using the fixed-effect model. According to the data, there was a more excellent ORR in the IPHC patients than that in the IPC patients [RR = 1.31, 95%CI (1.23, 1.38), *P* < 0.05]. Based on the scores from the NOS and the Jadad scale, literature is classified into low-quality and high-quality. In the subgroup of low quality, ORR in the IPHC patients was significantly higher than that in the IPC patients [RR = 1.67, 95%CI (1.30, 2.16), *P* < 0.05]. In the subgroup of high quality, the ORR in the IPHC patients was also significantly higher than that in the IPC patients [RR = 1.28, 95%CI (1.20, 1.35), *P* < 0.05]. Those results are shown in Fig. 2.

**Fig. 3:**
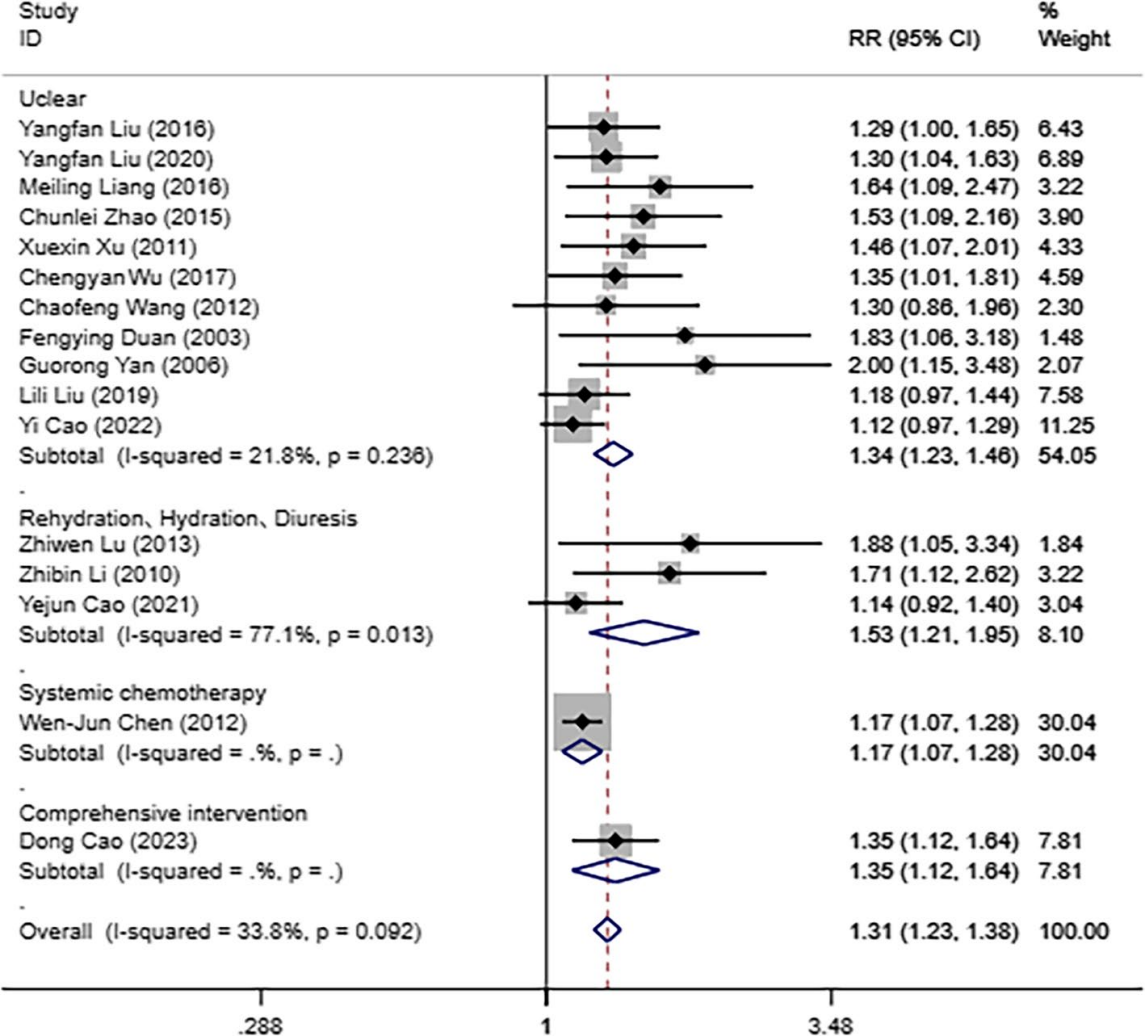
Forest plot for comparison of objective response rate for MPE patients in IPHC and IPC groups based on the combined with other treatments subgroup analysis. Note: MPE, malignant pleural effusion; IPHC, intrapleural perfusion with hyperthermic chemotherapy; IPC, intrapleural perfusion chemotherapy

Subgroup analysis can further determine whether the intrapleural perfusion is combined with other treatments. In the subgroup that included treatments such as rehydration, hydration, and diuresis, the ORR of the IPHC group was higher than that of the IPC group [RR = 1.53, 95%CI (1.21,1.95), *P* < 0.05]. In the subgroup that included systemic chemotherapy, the ORR of the IPHC group was higher than that of the IPC group [RR = 1.17, 95%CI (1.07,1.28), *P* < 0.05]. In the subgroup that included comprehensive intervention, the ORR of the IPHC group was higher than that of the IPC group [RR = 1.35, 95%CI (1.12,1.64), *P* < 0.05]. In the subgroup that was unclear therapy, the ORR of the IPHC group was higher than that of the IPC group [RR = 1.34, 95%CI (1.23,1.46), *P* < 0.05]. Those results are shown in Fig. 3.

We also conducted subgroup analyses based on the course of treatment and follow-up time. We found that the thermal perfusion chemotherapy regimen differed in each study. The results of the subgroup analyses showed that IPHC patients had higher ORR compared with IPC patients (*P* < 0.05). In the subgroup analysis of the origin of malignant pleural effusion, among the three subgroups of Non-small cell carcinoma, A variety of cancers, and Lung cancer, the results of the subgroup analyses indicated that patients with IPHC had a higher ORR compared with IPC patients (*P* < 0.05). However, in the two subgroups of small cell lung cancer and unclear carcinoma, there was no statistically significant difference in ORR between the two groups of patients (*P* > 0.05), as shown in Table 2.

**Table 2:** Subgroup analysis of ORR in patients with MPE after hyperthermic perfusion

Subgroup	Number of Articles	Heterogeneity test		Effect Model	Effect Size with 95% CI	Double-tailed test	
		I^2^(%)	P			Z	P
Course of treatment							
1week	1	-	-	Fixed effect	RR = 1.29,95%CI(1.01,1.65)	1.99	0.046
2weeks	4	42.2	0.585	Fixed effect	RR = 1.32,95%CI(1.16,1.50)	4.34	0.000
3weeks	5	0.0	0.650	Fixed effect	RR = 1.59,95%CI(1.33,1.90)	5.12	0.000
4weeks	2	2.2	0.312	Fixed effect	RR = 1.41,95%CI(1.15,1.73)	3.28	0.001
8weeks	1	-	-	Fixed effect	RR = 1.17,95%CI(1.07,1.28)	3.52	0.000
Unclear	2	45.4	0.160	Fixed effect	RR = 1.21,95%CI(1.06,1.38)	2.86	0.004
Origins of pleural effusion							
Non-small cell carcinoma	4	0.0	0.817	Fixed effect	RR = 1.28,95%CI(1.14,1.4)	4.09	0.000
A variety of cancers	7	50.7	0.058	Random effect	RR = 1.41,95%CI(1.19,1.67)	3.98	0.000
Small cell lung cancer	2	64.5	0.093	Random effect	RR = 1.31,95%CI(0.94,1.83)	1.61	0.108
Lung cancer	1	-	-	Fixed effect	RR = 1.35,95%CI(1.01,1.81)	2.00	0.046
Unclear carcinoma	2	86.7	0.006	Random effect	RR = 1.47,95%CI(0.65,3.28)	0.93	0.352
Follow-up time							
At least 4 weeks	14	22.9	0.206	Fixed effect	RR = 1.37,95%CI(1.27,1.48)	8.14	0.000
Unclear	2	0.0	0.801	Fixed effect	RR = 1.18,95%CI(1.08,1.27)	3.71	0.000

#### Improvement rate of life quality

**Fig. 4:**
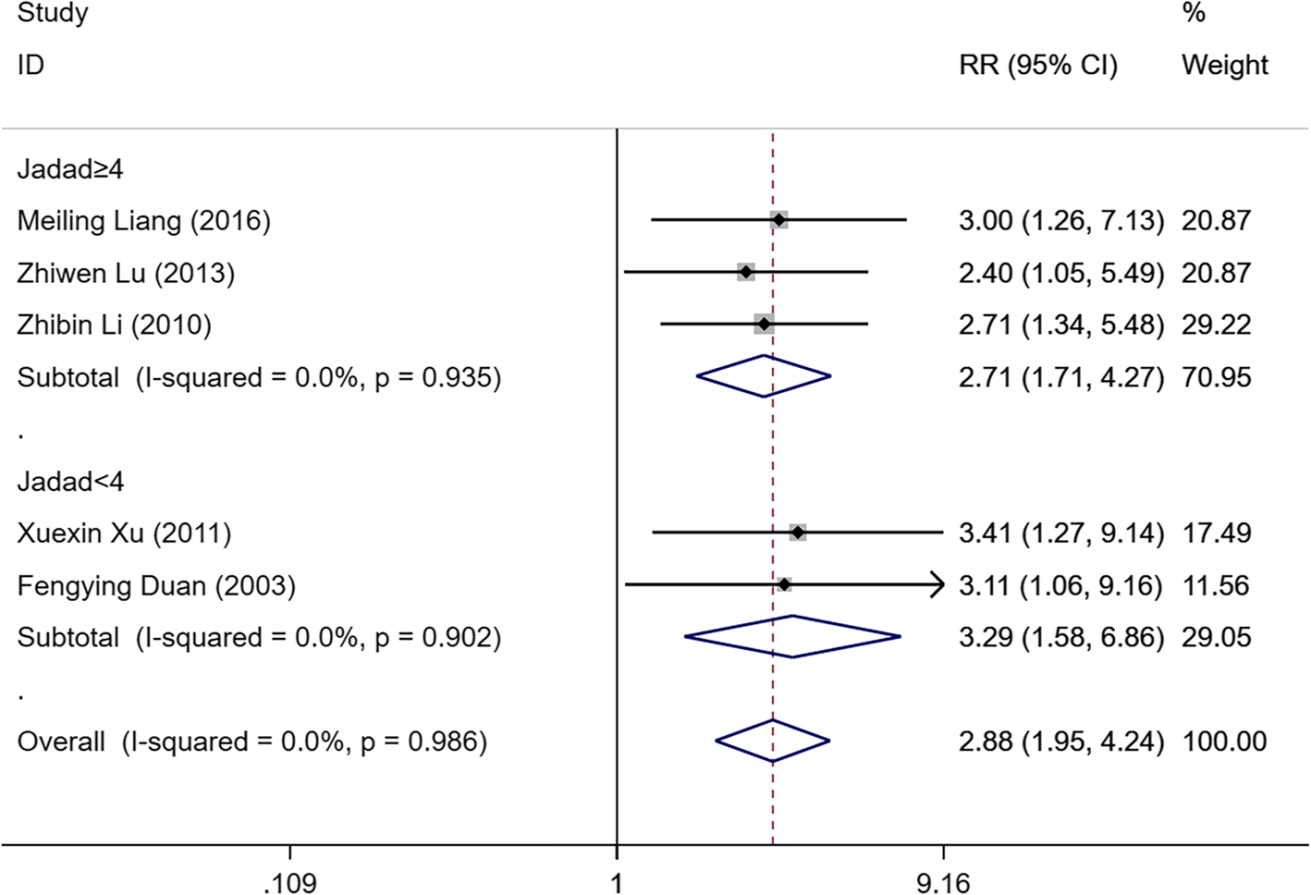
Forest plot for comparison of life quality improvement rates for MPE patients in IPHC and IPC groups based on the Jadad score subgroup analysis. Note: MPE, malignant pleural effusion; IPHC, intrapleural perfusion with hyperthermic chemotherapy; IPC, intrapleural perfusion chemotherapy

**Fig. 5:**
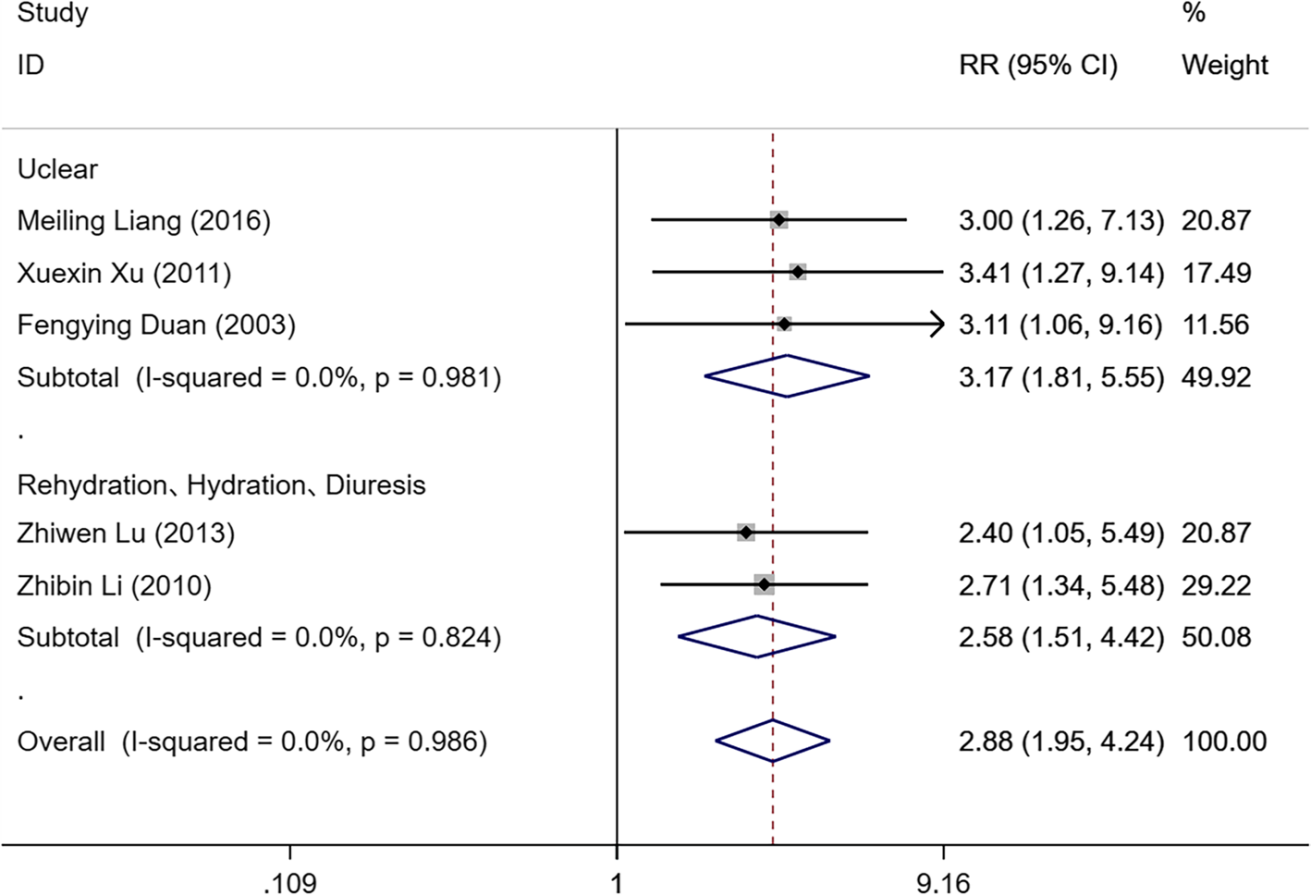
Forest plot for comparison of life quality improvement rates for MPE patients in IPHC and IPC groups based on the combined with other treatments subgroup analysis. MPE, malignant pleural effusion; IPHC, intrapleural perfusion with hyperthermic chemotherapy; IPC, intrapleural perfusion chemotherapy

Five studies [16,18,21–23] including 122 IPHC patients and 121 IPC patients, compared the improvement rate of life quality following IPHC or IPC for MPE. The results of the heterogeneity analysis for the literature showed no evidence of heterogeneity (I^2^ = 0.0% and *P* = 0.986). The fixed-effect model was used to combine the effect sizes. The results demonstrated a difference between the IPHC and IPC patients, and the improvement rate of life quality in the IPHC patients was higher than that in the IPC patients [RR = 2.88, 95%CI (1.95, 4.24), *P* < 0.05]. A subgroup analysis was performed according to the Jadad score. In the subgroup with a Jadad score ≥ 4, the improvement rate of life quality in the IPHC patients was significantly higher than that in the IPC patients [RR = 2.71, 95%CI (1.71, 4.27), *P* < 0.05]. In the subgroup with a Jadad score < 4, the improvement rate of life quality in the IPHC patients was also significantly higher than that in the IPC patients [RR = 3.29, 95%CI (1.58, 6.86), *P* < 0.05]. Those results are shown in Fig. 4. In the subgroup that included treatments with Rehydration, Hydration, and Diuresis, the improvement rate of life quality in the IPHC patients was significantly higher than that in the IPC patients [RR = 2.58, 95%CI (1.51, 4.42), *P* < 0.05]. In the subgroup where it was unclear whether other treatments were included or not, the improvement rate of life quality in the IPHC patients was also significantly higher than that in the IPC patients [RR = 3.17, 95%CI (1.81, 5.55), *P* < 0.05]. These results are displayed in Fig. 5.

#### Incidence of adverse reactions

The adverse reactions after intrapleural chemotherapy, which were extracted from the 16 included articles, mainly had asthenia, hepatic impairment, chest pain, leukopenia, gastrointestinal Reactions, and thrombocytopenia. An analysis was performed according to different adverse reactions (Table 3).

**Table 3:** Analysis of results of incidence of adverse reaction

Adverse reactions	Number of Articles	Heterogeneity test		Effect Model	Effect Size with 95% CI	Double-tailed test	
		I2(%)	P			Z	P
Asthenia	2	73.3	0.053	Random effect	RR = 0.70,95%CI(0.23,2.15)	0.61	0.539
Gastrointestinal Reactions	4	0.0	0.969	Fixed effect	RR = 0.76,95%CI(0.58,0.98)	2.14	0.032
Hepatic impairment	2	0.0	0.811	Fixed effect	RR = 0.83,95%CI(0.27,2.58)	0.32	0.752
Chest pain	5	5.2	0.377	Fixed effect	RR = 0.78,95%CI(0.61,1.00)	1.97	0.049
Jadad ≥ 4	3	0.0	0.592	Fixed effect	RR = 0.76,95%CI(0.59,0.97)	0.03	0.973
Jadad < 4	2	50.7	0.132	Fixed effect	RR = 0.98,95%CI(0.30,3.20)	2.18	0.029
Leukopenia	4	30.3	0.231	Fixed effect	RR = 0.82,95%CI(0.52,1.30)	0.83	0.405
Jadad ≥ 4	3	51.1	0.129	Fixed effect	RR = 0.78,95%CI(0.47,1.28)	1.00	0.319
Jadad < 4	1	-	-	Fixed effect	RR = 1.17,95%CI(0.37,3.69)	0.26	0.793
Thrombocytopenia	4	0.0	0.743	Fixed effect	RR = 0.96,95%CI(0.57,1.64)	0.13	0.895
Jadad ≥ 4	3	0.0	0.879	Fixed effect	RR = 1.05,95%CI(0.61,1.81)	0.17	0.861
Jadad < 4	1	-	-	Fixed effect	RR = 0.23,95%CI(0.01,4.38)	0.98	0.329

In the Asthenia subgroup, 73 MPE patients received IPHC, and 73 received IPC, according to 2 articles [14,19]. Asthenia was common in both IPHC and IPC groups. The results revealed no significant difference in the incidence rates of asthenia between the two groups [RR = 0.70, 95%CI (0.23, 2.15), *P* > 0.05].

Sixty-one patients had treatment for malignant pleural effusion using IPHC, and 61 patients received therapy of IPC in the subgroup of patients with hepatic impairment, according to two studies [19,23]. The results revealed no significant difference in the incidence rates of hepatic impairment in MPE patients between the two groups [RR = 0.83,95%CI(0.27,2.58), *P* > 0.05].

Four articles [14,19,21,23] described the incidence of leukopenia after IPHC and IPC for MPE, with 125 patients in the IPHC group and 125 patients in the IPC group. According to the results, there was little significant difference between the incidence rates of leukopenia between the two groups [RR = 0.82, 95%CI (0.52, 1.30), *P* > 0.05]. In the subgroup of Jadad score ≥ 4, there was no significant difference in the incidence rates of leukopenia between the IPHC and IPC groups [RR = 0.78, 95%CI (0.47, 1.28), *P* > 0.05]. In the subgroup of Jadad score < 4, there was no significant difference in the incidence rates of leukopenia between the IPHC group and IPC group [RR = 1.17, 95%CI (0.37, 3.69), *P* > 0.05)].

Four studies [14,19,21,23] in the subgroup of thrombocytopenia described the incidence of thrombocytopenia after IPHC and IPC for MPE, including 115 patients receiving IPHC and 117 receiving IPC. The results showed that the incidence rates of thrombocytopenia in MPE patients differed little between the two groups [RR = 0.96, 95%CI (0.57, 1.64), *P* > 0.05]. In the subgroup with a Jadad score ≥ 4, there was no significant difference in the incidence rates of thrombocytopenia between the IPHC and IPC groups [RR = 1.05, 95%CI (0.61, 1.81), *P* > 0.05]. In the subgroup with a Jadad score < 4, There was no significant difference in the incidence rates of thrombocytopenia between the IPHC and IPC groups [RR = 0.23, 95%CI (0.01, 4.38), *P* > 0.05].

Chest pain after IPHC and IPC for MPE was described in 5 studies [18,19,21,23,25], with 150 patients receiving IPHC and 149 receiving IPC. The results indicated that there was a lower incidence of chest pain in the IPHC group than in the IPC group [RR = 0.78, 95% CI (0.61, 1.00), *P* < 0.05]. In the subgroup with Jadad score < 4, there was no significant difference in the incidence rates of chest pain between the IPHC and IPC groups [RR = 0.98, 95%CI (0.30, 3.20), *P* > 0.05]. In the subgroup with a Jadad score ≥ 4, the incidence rate of chest pain of patients in the IPHC group was lower than that in the IPC group [RR = 0.76, 95%CI (0.59, 0.97), *P* < 0.05].

Five studies [16,25–28] in the subgroup of gastrointestinal responses described the incidence rate of gastrointestinal reactions after IPHC and IPC for malignant pleural effusion, with 108 cases in the IPHC group and 108 cases in the IPC group. Based on the studies, the incidence rate of gastrointestinal reactions of patients in the IPHC group was lower than that in the IPC group [RR = 0.76,95%CI(0.58,0.98), *P* < 0.05].

#### Publication bias

**Fig. 6:**
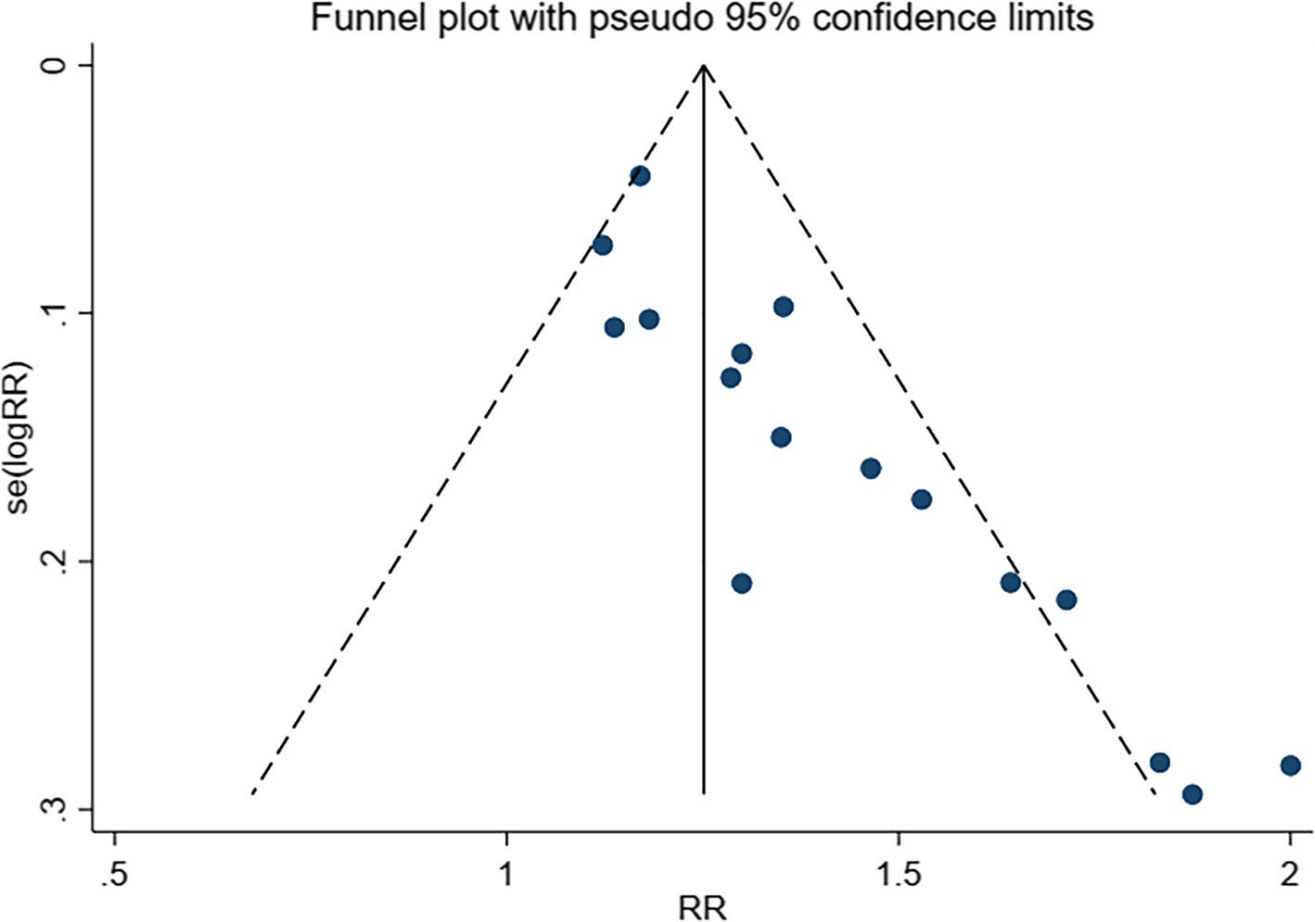
The funnel plot for evaluating the publication bias

In this meta-analysis, the included literature has an objective response rate of 16. A funnel plot was used to evaluate the publication bias (Fig. 6). The funnel plot showed that the distribution of each point was incomplete symmetry, suggesting particular publication bias. Some ongoing or gray literature may not be included in this meta-analysis. In addition, the literature, including the outcome measures of life quality improvement, adverse reactions, etc., was small. The Egger test was used to assess the publication bias. The results of the Egger test showed that chest pain (*P* = 0.989), leukopenia (*P* = 0.477), gastrointestinal reactions (*P* = 0.463), and thrombocytopenia (*P* = 0.407), suggesting that the literature, including the outcome measures of chest pain, leukopenia, gastrointestinal reactions, and thrombocytopenia were comprehensive, and did not have publication bias (*P* > 0.05). Due to the limited literature on asthenia and hepatic impairment, no publication bias detection was performed.

## Discussion

The obstruction of intrathoracic lymphatic return produces MPE, and it is mainly caused by a primary pleural tumor or the metastasis of other tumors to the pleura, which increases the permeability of the pleura, thereby resulting in the exudation and accumulation of lymph and tissue fluid in the thoracic cavity. The increase of pleural effusion restricts the mechanical expansion of the lungs, thereby affecting the functions of the heart and lungs, which often results in acute breathlessness and blood circulation failure. According to the International Association for the Study of Lung Cancer (IASLC), individuals with carcinomatous pleurisy had about 36% of 1-year survival rate [30]. To kill the tumor cells in the pleura, local chemotherapy is a commonly used treatment, especially perfusion chemotherapy, in which chemotherapeutic drugs are infused into the thoracic cavity and indwelled there. IPC is usually performed after thoracentesis and catheter drainage of the most pleural effusion, and chemotherapeutic drugs were injected into the thoracic cavity to control the production of pleural effusion effectively. According to studies, using recombinant human endostatin injection and IPC to treat MPE may help patients live longer overall and minimize the frequency of hospital stays [31], which suggests that IPC is a meaningful treatment.

With the continuous development of modern medicine, the model and method of hyperthermic perfusion therapy are also changing. At present, it generally includes the following four operation modes: (1) the perfusate is heated, and then perfused into the thoracic cavity; (2) the perfusate is heated by the endogenous field, and then perfused into the thoracic cavity; (3) the constant temperature water tank heats the perfusate, then perfused into the thoracic cavity, and last drained out of the thoracic cavity by the power pump; (4) the perfusate temperature with high accuracy was controlled, and then thoracic circulation and perfusion was performed [32]. In this study, the objective response rate of malignant pleural effusion in patients receiving IPHC treatment was significantly higher than in patients receiving IPC (*P* < 0.05), confirming that IPHC is more effective for MPE than IPC. IPHC may treat MPE by the following mechanisms: (1) Cancer cells are eliminated when heated to 41.0–45.0 °C for dozens of minutes by hyperthermic perfusion treatment, which also triggers tumor cell apoptosis by high temperature. The aberrant capillaries that sustain cancer cells make it difficult for them to store oxygen effectively. After heating, the properties of cancer cells cause them to dramatically slow down or inhibit their metabolism as well as the activities of enzymes necessary for cell division and DNA and RNA synthesis [33–35]; (2) Hyperthermic perfusion chemotherapy can stimulate the body’s immunity and promote the body’s anti-tumor ability; (3) IPHC can stimulate the anti-fibrinolytic effect in the pleural cavity, accelerate the condensation and deposition of fibrin and cellulose on the pleural surface, accelerate pleural fibrosis, and form an atretic pleural cavity; (4) IPHC can enhance the anti-tumor effect of chemotherapeutic drugs, and they jointly eliminate tumor cells [30]. Hyperthermia is a very effective tool for cancer treatment, mainly when it is used in combination with chemotherapy, radiotherapy, or immunotherapy, and they show a synergistic effect [36–38]. IPHC effectively keeps the relieved pleural effusion so that the patient’s lungs and heart have space for activities, which reduces the patient’s dyspnea and cardiac extrusion so that all parts of the patient’s body can obtain sufficient oxygen and blood supply, thereby significantly improving the patient’s life quality with the relatively free of movement.

IPHC also has the characteristics of chemotherapy and can cause a series of chemotherapy-related adverse effects in patients [39–42]. IPHC contained hyperthermia. Whether hyperthermia poses further damage to the patient has not been concluded. Studies have found that in the clinical treatment of MPE patients, the therapeutic efficacy of IPHC combined with recombinant human endostatin injection for MPE is improved, and the adverse reaction does not increase [43]. Similarly, this meta-analysis analyzed adverse reactions after IPHC or IPC treatment and found no significant differences in asthenia, thrombocytopenia, hepatic impairment, and leukopenia between the IPHC and IPC groups (*P* > 0.05). In addition, the incidence of chest pain and gastrointestinal reactions in MPE patients in the IPHC group was lower than that in the IPC group (*P* < 0.05); IPHC did not significantly increase the adverse reactions during the treatment, and to some extent, it may reduce the incidence of adverse reactions, which further demonstrated that IPHC is a relatively safe treatment. Many scholars have researched thoracic and thoracic hyperthermic perfusion, but no large-sample clinical study exists. This study collected the relevant literature, analyzed the clinical data of IPHC in treating MPE, and explored the efficacy of IPHC and IPC in treating MPE and the incidence of adverse reactions, thereby providing some reference value for the clinical treatment of MPE. This study found that IPHC was superior to IPC in MPE treatment, with relatively high safety. According to the results of this study, IPHC is recommended for treating MPE in clinical practice. However, this research has certain limitations, as follows: (1) The long-term treatment efficacy has to be investigated since there are no long-term follow-up data; (2) The strength of evidence in this systematic review needs to be improved owing to the small sample size and not very high quality of the controlled trials included. The results of asthenia and hepatic impairment are only reported in 2 literature, and the meta-analysis results are weak, which should be interpreted cautiously. The literature that met the inclusion criteria all came from Chinese scholars. Specific regional characteristics limit the universality of the research results; (3) There are differences in the type and dose of drugs, the number of taking IPHC, time interval, and temperature parameters, which can result in clinical heterogeneity, so that the results may be affected to some extent; (4) Since persistent or potential adverse effects in patients after IPHC treatment have not been reported in the original literature, ongoing or potential adverse effects of IPHC, indeed, are clinical concerns. In the future, we will try our best to perform much more studies on this problem.

## Conclusions

Compared with IPC, IPHC had a higher objective response rate without significantly increasing adverse reactions. Therefore, IPHC is effective and safe. However, the findings may be biased because of the included literature with non-standard research design and small sample size. Therefore, more high-quality, multi-center, large-sample, rigorously designed randomized controlled clinical studies are still needed for verification and evaluation.

## Data Availability

The datasets used and analyzed during the current study are available from the corresponding author upon reasonable request.
